# Chlorido{5,5′-dimeth­oxy-2,2′-[1,2-phenyl­enebis(nitrilo­methyl­idyne)]diphenolato-κ^4^ *O*,*N*,*N*′,*O*′}manganese(III)

**DOI:** 10.1107/S1600536808009835

**Published:** 2008-04-16

**Authors:** Naser Eltaher Eltayeb, Siang Guan Teoh, Suchada Chantrapromma, Hoong-Kun Fun, Rohana Adnan

**Affiliations:** a School of Chemical Science, Universiti Sains Malaysia, 11800 USM, Penang, Malaysia; b Department of Chemistry, Faculty of Science, Prince of Songkla University, Hat-Yai, Songkhla 90112, Thailand; c X-ray Crystallography Unit, School of Physics, Universiti Sains Malaysia, 11800 USM, Penang, Malaysia

## Abstract

In the title complex, [Mn(C_22_H_18_N_2_O_4_)Cl], the Mn^III^ centre is in a distorted square-pyramidal configuration, with the basal plane formed by the N_2_O_2_ donors of the tetra­dentate Schiff base dianion; the two phenolate O atoms and the two imine N atoms are each mutually *cis*. The chloride ion occupies the apical position. The dihedral angle between the two outer phenolate rings of the tetra­dentate Schiff base ligand is 16.44 (9)°. The central benzene ring makes dihedral angles of 10.64 (9) and 25.17 (10)° with the two outer phenolate rings. In the crystal structure, weak C—H⋯O and C—H⋯Cl inter­actions link the mol­ecules into wave-like face-to-face double layers along the *c* direction. A π–π inter­action involving the two outer phenolate rings is observed, the centroid–centroid distance being 3.743 (11) Å.

## Related literature

For values of bond lengths, see: Allen *et al.* (1987 ▶). For details of ring conformations, see: Cremer & Pople (1975 ▶). For related structures, see, for example: Eltayeb *et al.* (2008*a* ▶,*b* ▶); Habibi *et al.* (2007 ▶); Mitra *et al.* (2006 ▶). For the background to applications of manganese complexes, see, for example: Dixit & Srinivasan (1988 ▶); Glatzel *et al.* (2004 ▶); Lu *et al.* (2006 ▶).
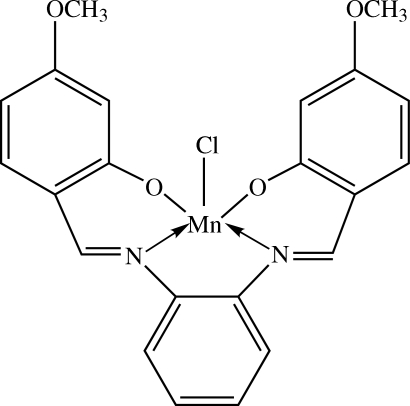


## Experimental

### 

#### Crystal data

[Mn(C_22_H_18_N_2_O_4_)Cl]
*M* *_r_* = 464.77
Orthorhombic, 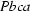

*a* = 13.7282 (2) Å
*b* = 15.0250 (2) Å
*c* = 19.2094 (3) Å
*V* = 3962.25 (10) Å^3^
*Z* = 8
Mo *K*α radiation
μ = 0.83 mm^−1^
*T* = 296 (2) K
0.44 × 0.42 × 0.11 mm

#### Data collection

Bruker SMART APEXII CCD area-detector diffractometer
Absorption correction: multi-scan (**SADABS**; Bruker, 2005 ▶) *T*_min_ = 0.708, *T*_max_ = 0.915
28689 measured reflections
5780 independent reflections
4072 reflections with *I* > 2σ(*I*)
*R*_int_ = 0.032

#### Refinement

*R*[*F* ^2^ > 2σ(*F* ^2^)] = 0.038
*wR*(*F* ^2^) = 0.095
*S* = 1.05
5780 reflections
273 parameters
H-atom parameters constrained
Δρ_max_ = 0.30 e Å^−3^
Δρ_min_ = −0.34 e Å^−3^

### 

Data collection: *APEX2* (Bruker, 2005 ▶); cell refinement: *APEX2*; data reduction: *SAINT* (Bruker, 2005 ▶); program(s) used to solve structure: *SHELXTL* (Sheldrick, 2008 ▶); program(s) used to refine structure: *SHELXTL*; molecular graphics: *SHELXTL*; software used to prepare material for publication: *SHELXTL* and *PLATON* (Spek, 2003 ▶).

## Supplementary Material

Crystal structure: contains datablocks global, I. DOI: 10.1107/S1600536808009835/is2286sup1.cif

Structure factors: contains datablocks I. DOI: 10.1107/S1600536808009835/is2286Isup2.hkl

Additional supplementary materials:  crystallographic information; 3D view; checkCIF report

## Figures and Tables

**Table 1:** Hydrogen-bond geometry (Å, °)

*D*—H⋯*A*	*D*—H	H⋯*A*	*D*⋯*A*	*D*—H⋯*A*
C7—H7*A*⋯Cl1^i^	0.93	2.81	3.7156 (19)	165
C21—H21*A*⋯O2^ii^	0.96	2.44	3.321 (2)	152

Symmetry codes: (i) 
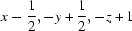
; (ii) 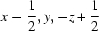.

**Fig. 1.**
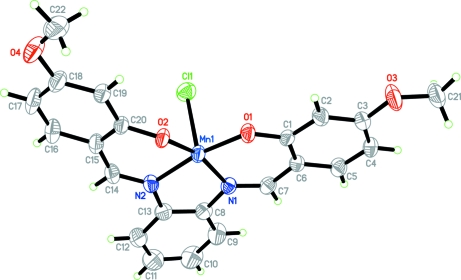
The asymmetric unit of (I), showing 50% probability displacement ellipsoids and the atomic numbering.

**Fig. 2.**
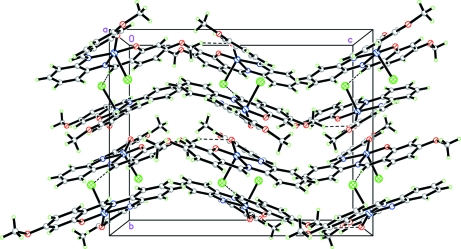
The crystal packing of (I), viewed along the a axis, showing the wave like face-to-face double layers along the c axis. C—H···O and C—H···Cl weak interactions are drawn as dashed lines.

## supplementary crystallographic information

### Comment

There has been considerable interest in Schiff base ligand containing oxygen and imine nitrogen atoms and their metal complexes due to their variety of applications such as manganese complexes with Schiff base ligands which have diverse range of applications in chemistry, biology, physics and advanced materials and are used in catalysis (Dixit & Srinivasan, 1988), as models for the oxygen-evolving complex of photosystem II (Glatzel *et al.*, 2004), and as single-molecule magnets (Lu *et al.*, 2006). We have previously reported the crystal structures of five coordinate Mn^III^ complexes with closely-related N_2_O_2_ donor Schiff base ligands, chlorido{6,6'-dimethyl-2,2'-[1,2-phenylenebis(nitrilomethylidene)]diphenolato- κ^4^*O*,*N*,*N*',*O*'}manganese(III) monohydrate (Eltayeb *et al.*, 2008*a*) and chlorido{5,5'-dimethyl-2,2'-[1,2- phenylenebis(nitrilomethylidene)]diphenolato- κ^4^*O*,*N*,*N*',*O*'}manganese(III) (Eltayeb *et al.*, 2008*b*). We report here the synthesis and structure of (I), Fig. 1, another five-coordinate Mn^III^ complex of a closely-related ligand.

In (I), the Mn^III^ complex shows a slightly distorted square-pyramidal geometry involving N1, N2, O1 and O2 atoms of the tetradentate Schiff base ligand as the basal plane. The two phenolic oxygen atoms and two imine nitrogen atoms are located in *cis* positions. The apical position is filled by the Cl^-^ ion. The Mn—O distances [Mn1—O1 = 1.8623 (12) Å, Mn1—O2 = 1.9067 (11) Å] and Mn—N distances [Mn1—N1 = 1.9859 (14) Å, Mn1—N2 = 1.9876 (14) Å] are in the same ranges as those observed in other related Mn^III^ complexes of N_2_O_2_ Schiff base ligands (Eltayeb *et al. *, 2008*a*,*b*; Habibi *et al.*, 2007; Mitra *et al.*, 2006). Other bond lengths and angles observed in the structure are also normal (Allen *et al.*, 1987). The basal bond angles are close to 90° [O1–Mn1–O2 = 92.82 (5)°, O1–Mn1–N1 = 93.06 (5)°, O2–Mn1–N2 = 90.13 (6)°] excepting for  the N–Mn–N angle is smaller than 90 ° [N1–Mn1–N2 = 81.68 (6)°]. The distorted square-pyramidal geometry of (I) can be reflected by the bond angles between the Cl^-^ ion and the atoms in the basal plane which are in the range 96.89 (4) to 97.16 (4)°. All these angle are close to the correspondence angles in the closely related structures (Eltayeb *et al.*, 2008*a*,*b*). Coordination of the N_2_O_2_ chelate ligand to the Mn^III^ ion results in the formation of an essentially planar five-membered ring (Mn1/N1/N2/C8/C13) and two six-membered rings; the Mn1/O1/N1/C1/C6/C7 ring is almost planar with the greatest deviation being -0.041 (1) Å for atom O1 whereas the Mn1/O2/N2/C14/C15/C20 ring adopts an envelope conformation with atom O2 displaced from the Mn1/N2/C14/C15/C20 plane by -0.276 (1) Å and with Cremer & Pople (1975) puckering parameters Q = 0.4418 (12)°, θ = 60.1 (2)° and φ = 20.2 (2)°. The dihedral angle between the two outer phenolate rings [C1–C6 and C15–C20] of the Schiff base ligand is 16.44 (9)°. The central benzene ring (C8–C13) makes dihedral angles of 10.64 (9) and 25.17 (10)° with the two outer phenolate rings. In addition one methoxy group is almost planarly attached to the (C15–C20) phenolate ring which can be indicated by the torsion angle C22–O4–C18–C19 = -3.4 (3)° whereas another methoxy group is slightly deviated from the mean plane of the C15–C20 phenolate ring, as shown by the torsion angle C21–O3–C3–C4 = 9.4 (3)°. The dihedral angles between the phenolate and benzene rings found in (I) are smaller than the corresponding angles found in a closely related structure (Eltayeb *et al.*, 2008*b*), showing that the Schiff base ligand in (I) is more flat due to the different substituents in the phenolate rings of the Schiff base ligand which are two methoxy groups in (I) but are two methyl groups in the same positions in (Eltayeb *et al.*, 2008*b*).

In the crystal packing (Fig. 2), weak C—H···O and C—H···Cl interactions (Table 1) link the molecules into wave like face-to-face double layers along the *c* direction. The crystal is stabilized by these weak C—H···O and C—H···Cl interactions. A π-π interaction was also observed in the crystal with the Cg_1_···Cg_2_^i^ distance of 3.7430 (11) Å [Cg_1_ and Cg_2_ are the centroids of the C1–C6 and C15–C20 phenolate rings, respectively; symmetry code: (i) 1-x, -y, 1-z].

### Experimental

The title compound was synthesized by adding 2-hydroxy-4-methoxybenzaldehyde (0.610 g, 4 mmol) to a solution of *o*-phenylenediamine (0.216 g, 2 mmol) in ethanol 95% (30 ml). The mixture was refluxed with stirring for half an hour. Manganese chloride tetrahydrate (0.394 g, 2 mmol) in ethanol (10 ml) was then added, followed by triethylamine (0.5 ml, 3.6 mmol). The mixture was refluxed at room temperature for three hours. A brown precipitate was obtained, washed with about 5 ml ethanol, dried, and then washed with copious quantities of diethylether. Brown single crystals of the title compound suitable for *x*-ray structure determination were recrystallized from methanol by slow evaporation of the solvent at room temperature after two weeks.

### Refinement

All H atoms were placed in calculated positions with d(C—H) = 0.93 Å and *U*_iso_(H) = 1.2*U*_eq_(C) for aromatic and CH, and with d(C—H) = 0.96 Å and *U*_iso_(H) = 1.5*U*_eq_(C) for CH_3_. A rotating group model was used for the methyl groups. The highest residual electron density peak is located at 0.70 Å from C2 and the deepest hole is located at 0.53 Å from Mn1.

### Crystal data

**Table:**

[Mn(C_22_H_18_N_2_O_4_)Cl]	*F*_000_ = 1904
*M**_r_* = 464.77	*D*_x_ = 1.558 Mg m^−^^3^
Orthorhombic, *P**b**c**a*	Mo *K*α radiation λ = 0.71073 Å
Hall symbol: -P 2ac 2ab	Cell parameters from 5780 reflections
*a* = 13.7282 (2) Å	θ = 2.1–30.0º
*b* = 15.0250 (2) Å	µ = 0.83 mm^−^^1^
*c* = 19.2094 (3) Å	*T* = 296 (2) K
*V* = 3962.25 (10) Å^3^	Block, brown
*Z* = 8	0.44 × 0.42 × 0.11 mm

### Data collection

**Table:**

Bruker SMART APEXII CCD area-detector diffractometer	5780 independent reflections
Radiation source: fine-focus sealed tube	4072 reflections with *I* > 2σ(*I*)
Monochromator: graphite	*R*_int_ = 0.032
Detector resolution: 8.33 pixels mm^-1^	θ_max_ = 30.0º
*T* = 296(2) K	θ_min_ = 2.1º
ω scans	*h* = −19→14
Absorption correction: multi-scan(*SADABS*; Bruker, 2005)	*k* = −21→16
*T*_min_ = 0.708, *T*_max_ = 0.915	*l* = −27→20
28689 measured reflections	

### Refinement

**Table:**

Refinement on *F*^2^	Secondary atom site location: difference Fourier map
Least-squares matrix: full	Hydrogen site location: inferred from neighbouring sites
*R*[*F*^2^ > 2σ(*F*^2^)] = 0.038	H-atom parameters constrained
*wR*(*F*^2^) = 0.095	*w* = 1/[σ^2^(*F*_o_^2^) + (0.0397*P*)^2^ + 1.0383*P*] where *P* = (*F*_o_^2^ + 2*F*_c_^2^)/3
*S* = 1.05	(Δ/σ)_max_ = 0.001
5780 reflections	Δρ_max_ = 0.30 e Å^−^^3^
273 parameters	Δρ_min_ = −0.34 e Å^−^^3^
Primary atom site location: structure-invariant direct methods	Extinction correction: none

### Special details

**Table:**

Geometry. All esds (except the esd in the dihedral angle between two l.s. planes) are estimated using the full covariance matrix. The cell esds are taken into account individually in the estimation of esds in distances, angles and torsion angles; correlations between esds in cell parameters are only used when they are defined by crystal symmetry. An approximate (isotropic) treatment of cell esds is used for estimating esds involving l.s. planes.
Refinement. Refinement of F^2^ against ALL reflections. The weighted R-factor wR and goodness of fit S are based on F^2^, conventional R-factors R are based on F, with F set to zero for negative F^2^. The threshold expression of F^2^ > 2sigma(F^2^) is used only for calculating R-factors(gt) etc. and is not relevant to the choice of reflections for refinement. R-factors based on F^2^ are statistically about twice as large as those based on F, and R- factors based on ALL data will be even larger.

### Fractional atomic coordinates and isotropic or equivalent isotropic displacement parameters (Å^2^)

**Table:**

	*x*	*y*	*z*	*U*_iso_*/*U*_eq_	
Mn1	0.475763 (18)	0.111700 (17)	0.483521 (14)	0.02817 (9)	
Cl1	0.54204 (4)	0.24455 (3)	0.42743 (3)	0.04223 (13)	
O1	0.40677 (8)	0.07486 (8)	0.40518 (6)	0.0327 (3)	
O2	0.58304 (8)	0.03537 (8)	0.46327 (6)	0.0303 (3)	
O3	0.16571 (10)	0.03819 (11)	0.23875 (7)	0.0550 (4)	
O4	0.92194 (10)	0.01474 (11)	0.41480 (9)	0.0595 (4)	
N1	0.35851 (10)	0.16427 (9)	0.52796 (7)	0.0289 (3)	
N2	0.53358 (11)	0.14005 (9)	0.57575 (8)	0.0314 (3)	
C1	0.31581 (12)	0.09053 (11)	0.38763 (9)	0.0285 (4)	
C2	0.28302 (13)	0.05942 (12)	0.32363 (9)	0.0357 (4)	
H2A	0.3262	0.0298	0.2944	0.043*	
C3	0.18761 (13)	0.07159 (13)	0.30249 (10)	0.0362 (4)	
C4	0.12079 (14)	0.11447 (12)	0.34578 (10)	0.0385 (4)	
H4A	0.0565	0.1224	0.3318	0.046*	
C5	0.15161 (13)	0.14455 (12)	0.40900 (10)	0.0366 (4)	
H5A	0.1068	0.1720	0.4383	0.044*	
C6	0.24892 (12)	0.13560 (11)	0.43162 (9)	0.0296 (4)	
C7	0.27287 (13)	0.16752 (11)	0.49861 (10)	0.0326 (4)	
H7A	0.2228	0.1933	0.5243	0.039*	
C8	0.37358 (13)	0.19174 (11)	0.59834 (9)	0.0334 (4)	
C9	0.30344 (16)	0.23117 (14)	0.64048 (11)	0.0485 (5)	
H9A	0.2425	0.2453	0.6225	0.058*	
C10	0.32499 (18)	0.24912 (16)	0.70897 (13)	0.0598 (6)	
H10A	0.2781	0.2755	0.7372	0.072*	
C11	0.41567 (18)	0.22843 (16)	0.73660 (11)	0.0576 (6)	
H11A	0.4288	0.2396	0.7833	0.069*	
C12	0.48587 (16)	0.19152 (14)	0.69479 (10)	0.0465 (5)	
H12A	0.5468	0.1779	0.7131	0.056*	
C13	0.46582 (13)	0.17445 (12)	0.62465 (9)	0.0343 (4)	
C14	0.62697 (13)	0.13753 (12)	0.58689 (10)	0.0358 (4)	
H14A	0.6489	0.1586	0.6296	0.043*	
C15	0.69814 (13)	0.10568 (11)	0.53996 (10)	0.0342 (4)	
C16	0.79809 (14)	0.11969 (13)	0.55549 (12)	0.0447 (5)	
H16A	0.8148	0.1502	0.5959	0.054*	
C17	0.86974 (15)	0.08976 (15)	0.51298 (13)	0.0503 (6)	
H17A	0.9347	0.1003	0.5238	0.060*	
C18	0.84519 (13)	0.04321 (13)	0.45301 (12)	0.0424 (5)	
C19	0.74893 (13)	0.02738 (12)	0.43567 (10)	0.0355 (4)	
H19A	0.7339	−0.0033	0.3950	0.043*	
C20	0.67467 (12)	0.05740 (11)	0.47896 (9)	0.0305 (4)	
C21	0.07328 (15)	0.05817 (18)	0.20853 (11)	0.0587 (6)	
H21A	0.0689	0.0310	0.1634	0.088*	
H21B	0.0664	0.1215	0.2040	0.088*	
H21C	0.0224	0.0355	0.2379	0.088*	
C22	0.90345 (17)	−0.03830 (18)	0.35536 (13)	0.0632 (7)	
H22A	0.9641	−0.0547	0.3340	0.095*	
H22B	0.8648	−0.0052	0.3228	0.095*	
H22C	0.8689	−0.0911	0.3690	0.095*	

### Atomic displacement parameters (Å^2^)

**Table:**

	*U*^11^	*U*^22^	*U*^33^	*U*^12^	*U*^13^	*U*^23^
Mn1	0.02087 (14)	0.03480 (14)	0.02885 (16)	0.00122 (10)	−0.00199 (11)	−0.00309 (10)
Cl1	0.0391 (3)	0.0402 (2)	0.0474 (3)	−0.00523 (19)	0.0018 (2)	0.0043 (2)
O1	0.0219 (6)	0.0452 (7)	0.0310 (7)	0.0052 (5)	−0.0026 (5)	−0.0052 (5)
O2	0.0183 (6)	0.0373 (6)	0.0352 (6)	0.0003 (5)	−0.0030 (5)	−0.0029 (5)
O3	0.0381 (8)	0.0883 (11)	0.0387 (8)	0.0124 (7)	−0.0166 (7)	−0.0130 (8)
O4	0.0255 (7)	0.0776 (11)	0.0754 (11)	0.0040 (7)	0.0088 (8)	0.0008 (9)
N1	0.0264 (7)	0.0297 (7)	0.0308 (8)	0.0000 (6)	0.0008 (6)	−0.0033 (6)
N2	0.0294 (8)	0.0345 (7)	0.0302 (8)	−0.0012 (6)	−0.0048 (7)	−0.0023 (6)
C1	0.0217 (8)	0.0319 (8)	0.0318 (9)	0.0001 (6)	−0.0006 (7)	0.0054 (7)
C2	0.0278 (9)	0.0495 (10)	0.0298 (10)	0.0055 (8)	−0.0011 (8)	−0.0024 (8)
C3	0.0308 (10)	0.0455 (10)	0.0324 (10)	−0.0005 (8)	−0.0061 (8)	0.0048 (8)
C4	0.0233 (9)	0.0491 (10)	0.0430 (11)	0.0036 (8)	−0.0066 (9)	0.0046 (9)
C5	0.0267 (9)	0.0444 (10)	0.0387 (11)	0.0080 (8)	−0.0006 (9)	−0.0007 (8)
C6	0.0253 (8)	0.0324 (8)	0.0310 (9)	0.0020 (7)	−0.0006 (8)	0.0013 (7)
C7	0.0262 (9)	0.0336 (8)	0.0379 (10)	0.0042 (7)	0.0033 (8)	−0.0014 (8)
C8	0.0338 (10)	0.0331 (9)	0.0331 (10)	−0.0020 (7)	0.0019 (8)	−0.0047 (7)
C9	0.0397 (11)	0.0594 (13)	0.0465 (13)	0.0036 (9)	0.0013 (10)	−0.0176 (10)
C10	0.0554 (15)	0.0755 (16)	0.0484 (14)	−0.0009 (12)	0.0121 (12)	−0.0266 (12)
C11	0.0626 (16)	0.0738 (15)	0.0363 (12)	−0.0106 (12)	0.0022 (12)	−0.0169 (11)
C12	0.0448 (12)	0.0584 (13)	0.0362 (11)	−0.0074 (10)	−0.0051 (10)	−0.0033 (9)
C13	0.0373 (10)	0.0343 (9)	0.0313 (9)	−0.0044 (8)	0.0010 (8)	−0.0042 (7)
C14	0.0341 (10)	0.0373 (9)	0.0359 (10)	−0.0020 (8)	−0.0118 (9)	−0.0014 (8)
C15	0.0260 (9)	0.0346 (9)	0.0422 (11)	−0.0019 (7)	−0.0083 (9)	0.0026 (8)
C16	0.0305 (10)	0.0445 (11)	0.0591 (14)	−0.0024 (8)	−0.0141 (10)	−0.0031 (10)
C17	0.0227 (10)	0.0528 (12)	0.0754 (17)	−0.0043 (9)	−0.0106 (11)	0.0021 (11)
C18	0.0241 (9)	0.0454 (10)	0.0578 (13)	0.0013 (8)	0.0033 (10)	0.0115 (10)
C19	0.0259 (9)	0.0415 (10)	0.0392 (10)	0.0013 (7)	−0.0007 (8)	0.0066 (8)
C20	0.0217 (8)	0.0321 (8)	0.0376 (10)	−0.0003 (7)	−0.0031 (8)	0.0072 (7)
C21	0.0392 (12)	0.0960 (18)	0.0410 (12)	0.0053 (12)	−0.0151 (11)	−0.0007 (12)
C22	0.0440 (13)	0.0847 (17)	0.0609 (15)	0.0148 (12)	0.0144 (12)	0.0119 (14)

### Geometric parameters (Å, °)

**Table:**

Mn1—O1	1.8623 (12)	C8—C9	1.390 (3)
Mn1—O2	1.9067 (11)	C9—C10	1.375 (3)
Mn1—N1	1.9859 (14)	C9—H9A	0.9300
Mn1—N2	1.9876 (14)	C10—C11	1.389 (3)
Mn1—Cl1	2.4440 (5)	C10—H10A	0.9300
O1—C1	1.3147 (19)	C11—C12	1.372 (3)
O2—C20	1.335 (2)	C11—H11A	0.9300
O3—C3	1.357 (2)	C12—C13	1.399 (3)
O3—C21	1.427 (2)	C12—H12A	0.9300
O4—C18	1.353 (2)	C14—C15	1.413 (3)
O4—C22	1.415 (3)	C14—H14A	0.9300
N1—C7	1.305 (2)	C15—C20	1.415 (3)
N1—C8	1.429 (2)	C15—C16	1.420 (3)
N2—C14	1.300 (2)	C16—C17	1.355 (3)
N2—C13	1.419 (2)	C16—H16A	0.9300
C1—C2	1.390 (2)	C17—C18	1.389 (3)
C1—C6	1.420 (2)	C17—H17A	0.9300
C2—C3	1.383 (2)	C18—C19	1.383 (3)
C2—H2A	0.9300	C19—C20	1.391 (3)
C3—C4	1.396 (3)	C19—H19A	0.9300
C4—C5	1.363 (3)	C21—H21A	0.9600
C4—H4A	0.9300	C21—H21B	0.9600
C5—C6	1.411 (2)	C21—H21C	0.9600
C5—H5A	0.9300	C22—H22A	0.9600
C6—C7	1.412 (2)	C22—H22B	0.9600
C7—H7A	0.9300	C22—H22C	0.9600
C8—C13	1.388 (3)		
			
O1—Mn1—O2	92.82 (5)	C8—C9—H9A	120.3
O1—Mn1—N1	93.06 (5)	C9—C10—C11	121.0 (2)
O2—Mn1—N1	162.37 (6)	C9—C10—H10A	119.5
O1—Mn1—N2	170.79 (6)	C11—C10—H10A	119.5
O2—Mn1—N2	90.13 (6)	C12—C11—C10	119.8 (2)
N1—Mn1—N2	81.68 (6)	C12—C11—H11A	120.1
O1—Mn1—Cl1	94.35 (4)	C10—C11—H11A	120.1
O2—Mn1—Cl1	96.53 (4)	C11—C12—C13	120.0 (2)
N1—Mn1—Cl1	99.58 (4)	C11—C12—H12A	120.0
N2—Mn1—Cl1	93.97 (4)	C13—C12—H12A	120.0
C1—O1—Mn1	129.57 (11)	C8—C13—C12	119.73 (18)
C20—O2—Mn1	122.17 (11)	C8—C13—N2	115.17 (16)
C3—O3—C21	119.09 (16)	C12—C13—N2	125.11 (17)
C18—O4—C22	118.41 (17)	N2—C14—C15	125.93 (17)
C7—N1—C8	121.91 (15)	N2—C14—H14A	117.0
C7—N1—Mn1	124.02 (12)	C15—C14—H14A	117.0
C8—N1—Mn1	113.85 (11)	C14—C15—C20	122.99 (16)
C14—N2—C13	123.23 (16)	C14—C15—C16	118.94 (18)
C14—N2—Mn1	122.28 (13)	C20—C15—C16	118.03 (18)
C13—N2—Mn1	113.96 (11)	C17—C16—C15	121.7 (2)
O1—C1—C2	118.30 (16)	C17—C16—H16A	119.1
O1—C1—C6	123.17 (16)	C15—C16—H16A	119.1
C2—C1—C6	118.50 (15)	C16—C17—C18	119.38 (18)
C3—C2—C1	121.45 (17)	C16—C17—H17A	120.3
C3—C2—H2A	119.3	C18—C17—H17A	120.3
C1—C2—H2A	119.3	O4—C18—C19	124.0 (2)
O3—C3—C2	115.20 (17)	O4—C18—C17	114.83 (17)
O3—C3—C4	124.24 (16)	C19—C18—C17	121.19 (19)
C2—C3—C4	120.56 (18)	C18—C19—C20	120.04 (18)
C5—C4—C3	118.68 (17)	C18—C19—H19A	120.0
C5—C4—H4A	120.7	C20—C19—H19A	120.0
C3—C4—H4A	120.7	O2—C20—C19	118.37 (16)
C4—C5—C6	122.45 (17)	O2—C20—C15	121.90 (16)
C4—C5—H5A	118.8	C19—C20—C15	119.63 (16)
C6—C5—H5A	118.8	O3—C21—H21A	109.5
C5—C6—C7	117.95 (16)	O3—C21—H21B	109.5
C5—C6—C1	118.32 (16)	H21A—C21—H21B	109.5
C7—C6—C1	123.62 (16)	O3—C21—H21C	109.5
N1—C7—C6	126.21 (16)	H21A—C21—H21C	109.5
N1—C7—H7A	116.9	H21B—C21—H21C	109.5
C6—C7—H7A	116.9	O4—C22—H22A	109.5
C13—C8—C9	119.98 (17)	O4—C22—H22B	109.5
C13—C8—N1	115.00 (16)	H22A—C22—H22B	109.5
C9—C8—N1	125.01 (17)	O4—C22—H22C	109.5
C10—C9—C8	119.4 (2)	H22A—C22—H22C	109.5
C10—C9—H9A	120.3	H22B—C22—H22C	109.5
			
O2—Mn1—O1—C1	169.80 (14)	C1—C6—C7—N1	3.4 (3)
N1—Mn1—O1—C1	6.44 (15)	C7—N1—C8—C13	172.65 (16)
Cl1—Mn1—O1—C1	−93.42 (14)	Mn1—N1—C8—C13	−2.17 (19)
O1—Mn1—O2—C20	147.01 (13)	C7—N1—C8—C9	−5.9 (3)
N1—Mn1—O2—C20	−103.7 (2)	Mn1—N1—C8—C9	179.32 (16)
N2—Mn1—O2—C20	−41.71 (13)	C13—C8—C9—C10	−2.8 (3)
Cl1—Mn1—O2—C20	52.30 (12)	N1—C8—C9—C10	175.59 (19)
O1—Mn1—N1—C7	−3.45 (15)	C8—C9—C10—C11	0.0 (4)
O2—Mn1—N1—C7	−112.8 (2)	C9—C10—C11—C12	1.6 (4)
N2—Mn1—N1—C7	−175.86 (15)	C10—C11—C12—C13	−0.3 (3)
Cl1—Mn1—N1—C7	91.49 (14)	C9—C8—C13—C12	4.1 (3)
O1—Mn1—N1—C8	171.25 (11)	N1—C8—C13—C12	−174.47 (16)
O2—Mn1—N1—C8	61.9 (2)	C9—C8—C13—N2	−175.66 (17)
N2—Mn1—N1—C8	−1.16 (11)	N1—C8—C13—N2	5.8 (2)
Cl1—Mn1—N1—C8	−93.82 (11)	C11—C12—C13—C8	−2.5 (3)
O2—Mn1—N2—C14	27.96 (14)	C11—C12—C13—N2	177.22 (19)
N1—Mn1—N2—C14	−167.72 (15)	C14—N2—C13—C8	165.17 (17)
Cl1—Mn1—N2—C14	−68.60 (14)	Mn1—N2—C13—C8	−6.68 (19)
O2—Mn1—N2—C13	−160.11 (12)	C14—N2—C13—C12	−14.6 (3)
N1—Mn1—N2—C13	4.21 (12)	Mn1—N2—C13—C12	173.56 (15)
Cl1—Mn1—N2—C13	103.33 (11)	C13—N2—C14—C15	−178.81 (17)
Mn1—O1—C1—C2	176.24 (12)	Mn1—N2—C14—C15	−7.6 (3)
Mn1—O1—C1—C6	−5.5 (2)	N2—C14—C15—C20	−11.7 (3)
O1—C1—C2—C3	178.41 (17)	N2—C14—C15—C16	170.58 (18)
C6—C1—C2—C3	0.0 (3)	C14—C15—C16—C17	179.18 (19)
C21—O3—C3—C2	−171.43 (18)	C20—C15—C16—C17	1.3 (3)
C21—O3—C3—C4	9.4 (3)	C15—C16—C17—C18	−0.8 (3)
C1—C2—C3—O3	179.73 (17)	C22—O4—C18—C19	−3.4 (3)
C1—C2—C3—C4	−1.1 (3)	C22—O4—C18—C17	175.86 (19)
O3—C3—C4—C5	179.50 (18)	C16—C17—C18—O4	−178.80 (19)
C2—C3—C4—C5	0.4 (3)	C16—C17—C18—C19	0.4 (3)
C3—C4—C5—C6	1.4 (3)	O4—C18—C19—C20	178.47 (17)
C4—C5—C6—C7	−178.81 (17)	C17—C18—C19—C20	−0.7 (3)
C4—C5—C6—C1	−2.4 (3)	Mn1—O2—C20—C19	−147.80 (13)
O1—C1—C6—C5	−176.64 (16)	Mn1—O2—C20—C15	35.8 (2)
C2—C1—C6—C5	1.7 (2)	C18—C19—C20—O2	−175.20 (16)
O1—C1—C6—C7	−0.5 (3)	C18—C19—C20—C15	1.3 (3)
C2—C1—C6—C7	177.84 (16)	C14—C15—C20—O2	−3.0 (3)
C8—N1—C7—C6	−174.68 (16)	C16—C15—C20—O2	174.78 (16)
Mn1—N1—C7—C6	−0.4 (2)	C14—C15—C20—C19	−179.30 (16)
C5—C6—C7—N1	179.55 (17)	C16—C15—C20—C19	−1.6 (3)

### Hydrogen-bond geometry (Å, °)

**Table:**

*D*—H···*A*	*D*—H	H···*A*	*D*···*A*	*D*—H···*A*
C7—H7A···Cl1^i^	0.93	2.81	3.7156 (19)	165
C21—H21A···O2^ii^	0.96	2.44	3.321 (2)	152

Symmetry codes: (i) *x*−1/2, −*y*+1/2, −*z*+1; (ii) *x*−1/2, *y*, −*z*+1/2.
